# Ultrasound-guided evaluation of airway difficulty in morbid obesity: Comparative predictive parameters

**DOI:** 10.6026/973206300212537

**Published:** 2025-08-31

**Authors:** Anoop Raveendran Nair, Ahmed Eltohamy, Nithya Krishnakumar

**Affiliations:** 1 Department of Anaesthesia, Pilgrim Hospital, UK; 2 Department of ENT, Kings Mill Hospital, UK

**Keywords:** Ultrasound, laryngoscopy, airway assessment and obesity

## Abstract

Ultrasound assessment of anterior neck soft tissue thickness is a promising tool for predicting difficult laryngoscopy, particularly in obese patients. Hence, we evaluated 40 obese individuals (BMI > 35) undergoing elective surgery, comparing ultrasonographic measurements at the hyoid bone (DSHB), thyrohyoid membrane (DSEM), and anterior commissure (DSAC) with conventional airway predictors. Difficult laryngoscopy, defined as Cormack-Lehane grade III-IV, was significantly associated with increased mean values of DSHB (p < 0.0001), DSEM (p< 0.0001) and DSAC (p< 0.002) respectively. Among conventional predictors, only the Modified Mallampati Score showed a significant correlation (p < 0.017). Ultrasonography of anterior neck soft tissues appears to be a reliable and independent predictor, potentially improving preoperative airway assessment in obese patients when combined with standard methods.

## Background:

Difficult laryngoscopy presents a significant challenge in airway management, particularly in obese patients undergoing general anaesthesia. Predicting intubation difficulty is essential for ensuring patient safety and optimizing anaesthetic techniques. Securing the airway through endotracheal intubation is critical to general anaesthesia and emergency care. However, unanticipated difficult laryngoscopy remains a major concern, with reported incidence rates ranging from 1.5% to 13% [1]. Conventional airway assessment tools such as the Modified Mallampati Score (MMS), thyromental distance (TMD) and neck mobility-have shown moderate sensitivity and significant inter-observer variability, limiting their reliability, especially in obese or uncooperative patients [2, 3]. Point-of-care ultrasound (POCUS) is a non-invasive, portable modality increasingly used in anaesthesia for vascular access and regional blocks and it holds potential in airway assessment as well [4]. Studies suggest increased anterior neck soft tissue thickness may impede pharyngeal structure displacement during laryngoscopy, thereby contributing to difficult glottic visualization [5, 6]. Difficult airway management is a significant concern in anaesthetic practice, particularly among obese patients. Individuals with a high body mass index (BMI) often present with anatomical and physiological variations that increase the complexity of airway management, potentially leading to complications during endotracheal intubation. Identifying reliable predictors of difficult intubation in obese patients can improve patient safety and guide perioperative strategies. By identifying reliable predictors, anaesthesiologists can refine airway management strategies and enhance perioperative outcomes. Therefore, it is of interest to assess the effectiveness of ultrasound-measured anterior neck soft tissue thickness (at the hyoid bone, thyrohyoid membrane and anterior commissure) in predicting difficult laryngoscopy in obese patients (BMI >35) and to compare its predictive value with conventional clinical parameters, including age, sex, BMI, Modified Mallampati Score (MMS), upper teeth pathology, mouth opening, neck mobility, neck circumference and thyromental distance (TMD).

## Methodology:

This prospective observational study was conducted to evaluate ultrasound measurements of anterior neck soft tissue as potential indicators of difficult laryngoscopy in obese patients. The study aimed to establish correlations between ultrasound-measured anterior neck thickness at specific anatomical landmarks and laryngoscopic view assessments, helping anaesthesiologists anticipate airway management challenges more effectively. This study was carried out in the Department of Anaesthesiology at Amala Institute of Medical Sciences, Thrissur, over an 18-month period from January 2016 to June 2017. The prospective observational design allowed for the collection of real-time data during perioperative airway assessments. A total of 40 patients aged 18 years and older, with a BMI exceeding 35 kg/m^2^, scheduled for elective surgeries under general anaesthesia with endotracheal intubation, were enrolled in the study. Patients were classified as American Society of Anesthesiologists (ASA) I or II, ensuring a homogeneous study population with minimal confounding factors related to systemic illnesses. Written informed consent was obtained from all participants prior to enrollment. To minimize confounding variables, patients meeting specific exclusion criteria were omitted from the study. These included refusal to participate, pregnancy, upper airway pathology, cervical spine injuries, presence of a full stomach at the time of surgery, hiatus hernia and gastroesophageal reflux disease (GERD). The sample size was calculated based on prior research by Wu *et al.* which assessed anterior neck soft tissue thickness at the thyrohyoid membrane (DSEM) as a predictor of difficult laryngoscopy. Using the mean and standard deviation from that study, a minimum sample size of 27 participants was required for statistical relevance. However, a total of 40 patients were included to enhance data robustness. Consecutive sampling methodology was adopted to ensure unbiased patient recruitment. Preoperative airway assessments were conducted for all patients, including evaluations for a history of obstructive sleep apnea (OSA), presence of abnormal upper teeth (protrusion, crowding, missing teeth affecting laryngoscopy), thyromental distance less than 6 cm, limited jaw mobility (inter-incisor gap less than 4 cm), restricted neck movement (less than 90 degrees), Modified Mallampati Score (MMS) classification and neck circumference measured at the level of the thyroid cartilage. These parameters were meticulously recorded, as they serve as established predictors of difficult airway management. A VENUE 40 linear probe (5.0 MHz) was used to obtain ultrasound measurements of anterior neck soft tissue thickness at three critical anatomical landmarks: the hyoid bone (DSHB), the thyrohyoid membrane (DSEM) and the anterior commissure (DSAC). Each measurement was averaged from three reference points: the midline and 1.5 mm lateral positions on both sides. These values were analyzed to determine correlations with intubation difficulty. Standard monitoring and preoxygenation were performed before the induction of anaesthesia. The following agents were used: fentanyl (1.5 mcg/kg) for analgesia, propofol (titrated to loss of verbal response) for induction and vecuronium (0.1 mg/kg) as a neuromuscular blocker. Laryngoscopy was performed using a size 3 Macintosh blade in the sniffing position, ensuring optimal visualization of the vocal cords. The anaesthesiologist performing the laryngoscopy was blinded to ultrasound results to prevent bias. The Cormack-Lehane grading system was employed to classify the laryngoscopic view. Grade I indicated full visualization of the vocal cords, Grade II indicated partial visualization, Grade III indicated only the epiglottis was visible (suggesting difficult intubation) and Grade IV indicated that neither the vocal cords nor the epiglottis were visible (suggesting severely difficult intubation). Grades III and IV were considered indicative of difficult laryngoscopy. If intubation attempts failed after three tries, the difficult airway management protocol was activated, involving adjunct airway devices and alternative intubation techniques. Data were recorded in Microsoft Excel and subsequently analyzed using SPSS v16. Continuous variables were expressed as mean ± standard deviation (SD), while categorical data were presented as percentages. Comparative analysis was performed using two-sided Student's t-tests for continuous data and chi-square tests for categorical data. A p-value of less than 0.05 was considered statistically significant. Ethical approval for the study was granted by the Institutional Research and Ethics Committee prior to initiation. All procedures adhered to international ethical guidelines for human research, ensuring patient safety and confidentiality. Ultrasound measurements of anterior neck soft tissue offer valuable insights into predicting difficult laryngoscopy in obese patients undergoing general anaesthesia. By identifying correlations between soft tissue thickness and airway visualization, anaesthesiologists can refine airway management strategies, improving patient outcomes in high-risk surgical scenarios. Therefore it is of interest to show additional imaging parameters and expand patient sample sizes to validate these findings further.

## Observations and Results:

The study was conducted on 40 obese patients (BMI >35) scheduled for elective surgeries under general anaesthesia with endotracheal intubation. The mean age of the patients in the easy laryngoscopy group was 33.90 ± 14.08 years, while that of the difficult laryngoscopy group was higher at 42.60 ± 3.93 years (Table 1 - see PDF). Females constituted 60% of the study population and males 40% (Figure 1 - see PDF). The mean BMI was also higher in the difficult laryngoscopy group and the difference was statistically significant (p = 0.007), reinforcing the possibility of a relationship between higher BMI and increased airway difficulty (Table 1 - see PDF).

Table 2 (see PDF) shows the data analysis of the the Modified Mallampati Score which was assessed for 40 patients with a BMI greater than 35 kg/m^2^, scheduled for elective surgeries under general anaesthesia. The score categorizes patients based on visibility of the oropharyngeal structures when they open their mouth and protrude their tongue. A lower score suggests an easier intubation, while a higher score is associated with greater difficulty. Among the traditional predictors evaluated, only the Modified Mallampati Score (MMS) showed a statistically significant correlation with difficult laryngoscopy (p = 0.017). As detailed, 12.5% of patients had MMS Grade I, 52.5% had Grade II and 35% had Grade III. All MMS Grade I patients had easy laryngoscopies, while the majority of those with Grade III (9 out of 14) experienced difficult laryngoscopy. Out of the total participants, five individuals (12.5%) were classified as Mallampati Class I, indicating complete visibility of the soft palate, uvula, fauces and tonsillar pillars. This classification is generally associated with a straightforward intubation process with minimal risk of complications. A majority of patients, total 21 (52.5%), fell under Mallampati Class II, where the soft palate, fauces and uvula were visible, but the tonsillar pillars were obscured. While this score still suggests relatively uncomplicated airway management, it may indicate a slightly increased risk compared to Class I patients. Fourteen patients (35%) were classified as Mallampati Class III, in which only the soft palate and base of the uvula were visible. This classification is associated with a significant likelihood of difficult intubation, requiring additional airway precautions and possibly alternative techniques to ensure successful airway management. The distribution highlights that while a majority of the study population presented with relatively favourable airway anatomy (Class I and II making up 65%), a notable proportion of patients (35%) exhibited anatomical features linked to difficult laryngoscopy (Class III). Since none of the patients in the study fell into Mallampati Class IV, which is associated with the most severe airway obstruction-where only the soft palate is visible-this suggests that extreme difficulty in intubation may not have been a predominant issue within this specific sample.

Table 3 (see PDF) describes the frequency and percentage distribution of TMD scores which were analyzed among 40 patients, providing insight into airway characteristics in individuals with a high BMI undergoing general anaesthesia. The findings indicate that 5 patients (12.5%) had a TMD score of 0, suggesting normal anatomical conditions with a lower likelihood of difficult airway management. Meanwhile, 35 patients (87.5%) had a TMD score of 1, signifying an increased risk for difficult laryngoscopy and intubation. The high prevalence of TMD score 1 (87.5%) underscores the importance of preoperative airway assessment and preparedness for alternative airway management strategies. Patients with a reduced TMD often exhibit posterior displacement of the larynx, limited jaw mobility, or altered neck extension, all of which contribute to complications during direct laryngoscopy. Table 4 (see PDF), describes the frequency and percentage distribution of OSA scores were analyzed among 40 patients with a BMI greater than 35 kg/m^2^. The results indicate that 18 patients (45.0%) did not exhibit characteristics of OSA, classified with an OSA score of 0. These individuals likely had relatively normal upper airway anatomy, leading to a reduced risk of difficult intubation. Conversely, 22 patients (55.0%) were classified with an OSA score of 1, signifying the presence of obstructive sleep apnea-related airway characteristics. These patients were at a higher risk for airway obstruction and difficult intubation due to increased soft tissue thickness, narrowed pharyngeal airway space and decreased airway compliance. The prevalence of OSA within this population underscores the importance of preoperative airway evaluation and risk stratification. Patients with OSA often require tailored anaesthetic approaches, including preoxygenation strategies, careful selection of sedatives and muscle relaxants and consideration of advanced airway techniques such as video laryngoscopy or fiber-optic intubation. The most important findings emerged from ultrasound-based assessments of anterior neck soft tissue thickness, measured at three anatomical landmarks: hyoid bone (DSHB), thyrohyoid membrane (DSEM) and anterior commissure (DSAC). These measurements demonstrated strong and statistically significant associations with difficult laryngoscopy. Table 5 (see PDF), describes the comparison of patients with and without difficult laryngoscopy highlights significant differences in several airway assessment parameters. Neck circumference was slightly higher in patients with difficult laryngoscopy (47.05 ± 4.478 cm) compared to those with normal laryngoscopic views (44.95 ± 3.531 cm), though this difference did not reach statistical significance (p = 0.108). This suggests that while neck circumference may contribute to airway difficulty, it is not a definitive predictor on its own. Ultrasound-based soft tissue measurements provided more substantial correlations with difficult laryngoscopy. The distance from the hyoid bone (DSHB) showed a statistically significant increase in difficult laryngoscopy cases (1.6790 ± 2.2466 cm) compared to non-difficult cases (1.2980 ± 1.0948 cm), with a highly significant p value of 0.0001. Similarly, the thickness at the thyrohyoid membrane (DSE*M) was greater in patients with difficult laryngoscopy (2.1010 ± 2.2541 cm) than in those with normal intubation conditions (1.5335 ± 1.5988 cm), also with a p value of 0.0001, reinforcing its predictive reliability. Measurements at the anterior commissure (DSAC) demonstrated a significant difference as well, with difficult laryngoscopy cases showing increased thickness (1.3035 ± 1.4964 cm) compared to non-difficult cases (1.1695 ± 1.0123 cm). Though the p value (0.002) indicates statistical significance, it is less pronounced compared to DSHB and DSEM.

Although neck circumference was slightly greater in the difficult laryngoscopy group (47.05 ± 4.478 cm) compared to the easy group (44.95 ± 3.531 cm), this difference did not reach statistical significance (p = 0.108) (Table 5 - see PDF). This indicates that general anthropometric measurements may not be as reliable as localized sonographic evaluations in predicting airway difficulty in obese patients. The highly significant correlations observed with DSHB and DSEM indicate their potential as valuable preoperative assessment tools. Integrating these measurements into airway screening protocols could enhance the accuracy of intubation difficulty predictions, allowing anaesthesiologists to adopt advanced airway techniques when necessary. In summary, the findings strongly support the use of ultrasound-measured anterior neck soft tissue thickness, particularly at the level of the thyrohyoid membrane (DSEM), as a sensitive and specific predictor of difficult laryngoscopy. Compared to conventional assessment tools, ultrasound provided greater predictive accuracy. While the Modified Mallampati Score retained some value as a standalone clinical test, other traditional parameters such as TMD, OSA history and neck circumference showed no meaningful correlation with difficult airway outcomes. These results underscore the clinical utility of incorporating point-of-care ultrasonography into routine preoperative airway assessments, especially for high-risk populations such as the morbidly obese.

## Discussion:

This study aimed to evaluate the feasibility and predictive accuracy of anterior neck soft tissue measurements using ultrasound (US) in identifying difficult laryngoscopy in obese patients and to compare these findings with conventional clinical screening tools. Our findings demonstrated a strong positive correlation between difficult laryngoscopy and increased anterior neck soft tissue thickness at three specific anatomical levels-hyoid bone (DSHB), thyrohyoid membrane (DSEM) and anterior commissure (DSAC). The measurements at all three levels were significantly higher in the difficult laryngoscopy group, with p-values of <0.0001 for DSHB and DSEM and <0.002 for DSAC, indicating that sonographic measurement of pretracheal soft tissue is a statistically and clinically significant predictor of airway difficulty. Among the traditional predictors evaluated, only the Modified Mallampati Score (MMS) showed a statistically significant association with difficult laryngoscopy (p < 0.17), albeit weaker than the ultrasound findings. Other standard indices-including thyromental distance (TMD), mouth opening, neck circumference, limited neck mobility, abnormal dentition and history of obstructive sleep apnea (OSA)-failed to demonstrate independent predictive value in our cohort. These findings are consistent with previous studies by Ezri *et al.*, Adhikari *et al.* and Wu *et al.* who also reported that anterior neck soft tissue thickness measured at the hyoid bone, thyrohyoid membrane and vocal cord levels correlates strongly with difficult airway outcomes [7, 4 and 10]. Ultrasound assessment of pre-epiglottic tissue thickness at the level of the thyrohyoid membrane may be useful to predict restricted/difficult direct laryngoscopy and difficult intubation [5]. Wu *et al.* in particular, established significant intercorrelations among these ultrasound parameters and supported their inclusion as independent predictors [10]. In contrast, the findings of Komatsu *et al.* who reported thinner pretracheal soft tissue in the difficult laryngoscopy group were not in agreement with ours [6]. This discrepancy may be attributed to differences in ethnic backgrounds between study populations, as their cohort consisted mainly of Caucasians and African Americans, while our population was South Asian. Furthermore, variations in ultrasound techniques and the anatomical landmarks selected for measurement may have contributed to the divergence in results. It is also worth noting that the 2 mm difference in pretracheal soft tissue reported by Komatsu *et al.* although statistically significant [6], may lack clinical relevance compared to the larger differences observed in our study and those by Ezri *et al.* [7]. Interestingly, while several studies, such as that by Whittle *et al.* noted a higher tendency for difficult intubation in males due to increased fat deposition in the submandibular region, our findings contrast this, with females comprising 75% of the difficult laryngoscopy group. Additionally, among patients with a history of OSA, 59% were female. This gender distribution challenges prior assumptions and emphasizes the importance of individualized assessment over generalized demographic trends. Collectively, these findings suggest that anterior neck soft tissue thickness measured by ultrasound provides a more sensitive and specific method for predicting difficult laryngoscopy in obese patients than traditional bedside assessments.

The non-invasive nature of ultrasound, its repeatability and its real-time applicability further support its integration into routine preoperative airway evaluation protocols, especially in high-risk populations. As supported by multiple studies and reaffirmed by our data, a multimodal approach that combines ultrasound parameters with selective clinical predictors such as MMS may yield the most reliable model for anticipating airway challenges [4, 5 and 10]. Morbid obesity is linked to difficult laryngoscopy and intubation, with standard predictors like MMS, TMD and neck mobility showing moderate sensitivity [8]. These findings emphasize the importance of ultrasound-based preoperative airway assessment in obese patients, as soft tissue thickness at specific anatomical landmarks correlates strongly with difficult laryngoscopy. Traditional predictors such as Modified Mallampati Score and thyromental distance (TMD) remain useful, but ultrasound imaging adds an additional layer of precision in identifying anatomical contributors to intubation difficulties [9]. For clinical practice, incorporating routine ultrasound evaluation of anterior neck soft tissue measurements can improve airway risk stratification and guide anaesthetic planning. The ultrasound method as a predictor of difficult intubation is promising in anesthetic practice when used according to standardized measurements evaluation and cutoff values [11]. Patients with increased soft tissue thickness at DSHB, DSEM and DSAC should be considered at higher risk for difficult intubation, warranting the use of advanced airway management techniques such as video laryngoscopy, fiber-optic intubation, or supraglottic airway devices to enhance intubation success and patient safety. A positive Mallampati test alone is insufficient for predicting difficult intubation in obese patients. This study hypothesized that ultrasound measurement of pretracheal soft tissue could be a better predictor. Ultrasound was used to measure neck soft tissue at the vocal cords, thyrohyoid membrane and anterior commissure in 40 morbidly obese patients. While MMS showed significance, other standard predictors were not independent indicators of difficult laryngoscopy. The study concluded that increased pretracheal soft tissue thickness is a reliable predictor of difficult laryngoscopy in obese patients.

## Conclusion:

This study concluded that anterior neck soft tissue thickness measured by ultrasound at the hyoid bone, thyrohyoid membrane and anterior commissure levels is are independent predictor of difficult laryngoscopy. Additionally, common used screening tests for difficult intubation, when used alone, demonstrated only moderate predictive power. However, combining these traditional screening tests with ultrasound measurements could enhance the ability to predict difficult laryngoscopy. The ultrasound technique proved to be safe, effective and non-invasive. The main limitations of the study include the relatively small sample size, which could be improved with a larger population for clearer results and the overrepresentation of patients with a BMI >40 (22 out of 40), where a more balanced distribution could yield more robust conclusions. Moreover, the study was conducted solely on an Indian population and a larger multicenter study across different ethnic groups could provide more generalizable findings.

## Funding statement:

No external funding was received for this study.

## Author contributions:

Frist author: Conceptualization, Methodology, Writing - Original Draft

Second author: Data Analysis, Writing - Review and Editing

third author Name: Data Collection, Literature Review
